# Obeticholic acid, a selective farnesoid X receptor agonist, regulates bile acid homeostasis in sandwich‐cultured human hepatocytes

**DOI:** 10.1002/prp2.329

**Published:** 2017-06-21

**Authors:** Yuanyuan Zhang, Jonathan P. Jackson, Robert L. St. Claire, Kimberly Freeman, Kenneth R. Brouwer, Jeffrey E. Edwards

**Affiliations:** ^1^ Intercept Pharmaceuticals Inc. San Diego California; ^2^ Qualyst Transporter Solutions Durham North Carolina

**Keywords:** bile acid, Cholestatic disease, Farnesoid X receptor, FXR, homeostasis, obeticholic acid, OCA

## Abstract

Farnesoid X receptor (FXR) is a master regulator of bile acid homeostasis through transcriptional regulation of genes involved in bile acid synthesis and cellular membrane transport. Impairment of bile acid efflux due to cholangiopathies results in chronic cholestasis leading to abnormal elevation of intrahepatic and systemic bile acid levels. Obeticholic acid (OCA) is a potent and selective FXR agonist that is 100‐fold more potent than the endogenous ligand chenodeoxycholic acid (CDCA). The effects of OCA on genes involved in bile acid homeostasis were investigated using sandwich‐cultured human hepatocytes. Gene expression was determined by measuring mRNA levels. OCA dose‐dependently increased fibroblast growth factor‐19 (FGF‐19) and small heterodimer partner (SHP) which, in turn, suppress mRNA levels of cholesterol 7‐alpha‐hydroxylase (CYP7A1), the rate‐limiting enzyme for de novo synthesis of bile acids. Consistent with CYP7A1 suppression, total bile acid content was decreased by OCA (1 *μ*mol/L) to 42.7 ± 20.5% relative to control. In addition to suppressing de novo bile acids synthesis, OCA significantly increased the mRNA levels of transporters involved in bile acid homeostasis. The bile salt excretory pump (BSEP), a canalicular efflux transporter, increased by 6.4 ± 0.8‐fold, and the basolateral efflux heterodimer transporters, organic solute transporter α (OST_*α*_) and OST_*β*_ increased by 6.4 ± 0.2‐fold and 42.9 ± 7.9‐fold, respectively. The upregulation of BSEP and OST_*α*_ and OST_*β*,_ by OCA reduced the intracellular concentrations of d_8_‐TCA, a model bile acid, to 39.6 ± 8.9% relative to control. These data demonstrate that OCA does suppress bile acid synthesis and reduce hepatocellular bile acid levels, supporting the use of OCA to treat bile acid‐induced toxicity observed in cholestatic diseases.

**Abbreviations**
BAAT: bile Acid‐CoA:Amino Acid N‐Acyltransferase
BACS: bile acid acyl‐CoA synthetase
BCRP: breast cancer resistance protein
BEI: biliary excretion index
BSEP: bile salt excretory pump
CA: cholic acid
CCM: cell culture medium
CDCA: chenodeoxycholic acid
CYP7A1: cholesterol 7‐alpha‐hydroxylase
CYP7B1: Cytochrome P450 family 7 subfamily B member 1
CYP8B1: cytochrome P450 family 8 subfamily B member 1
d_8_‐TCA: deuterium‐labeled sodium taurocholate
FDA: food and drug Administration
FXR: farnesoid X receptor
ICC: intracellular concentrations
MRP2: multidrug resistance‐associated protein 2
MRP3 and MRP4: multidrug resistance‐associated protein 3 and 4
NR1H4: nuclear receptor subfamily 1 group H Member 4
NTCP: sodium‐taurocholate cotransporting polypeptide
OATP1B1, OATP1B3, OATP2B1: organic anion transporting polypeptides 1B1, 1B3, 2B1; OST_*α*_, organic solute transporter *α*
OCA: obeticholic acid
OST_*β*_: organic solute transporter *β*
PBC: primary biliary cholangitis
PFIC2: progressive familial intrahepatic cholestasis type 2
P‐gp: P‐glycoprotein
PSC: primary sclerosing cholangitis
QC: quality control
qRT‐PCR: quantitative real‐time polymerase chain reaction
SCHH: sandwich‐cultured human hepatocytes
SHP: small heterodimer partner

## Introduction

Bile acids are synthesized from cholesterol metabolism exclusively in the liver (Russell and Setchell 1992). In addition to the conventional roles in digestion and absorption of lipid and lipid soluble nutrients in the small intestine, bile acids are also signaling molecules regulating hepatic lipid, glucose, and energy homeostasis (Watanabe et al. 2006; Thomas et al. 2009; Teodoro et al. 2011; Prawitt et al. 2014). Over a decade of research has proven Farnesoid X receptor (FXR) a key regulator in maintaining bile acid homeostasis (Forman et al. 1995; Parks et al. 1999; Wang et al. 1999). FXR is a ligand‐activated nuclear receptor, predominantly expressed in the liver, intestine, kidney, and adrenal gland (Forman et al. 1995; Parks et al. 1999; Wang et al. 1999). Bile acids are endogenous ligands for FXR (Forman et al. 1995; Parks et al. 1999; Wang et al. 1999). Rising bile acid concentrations activate FXR which in turn induces the transcription of small heterodimer partner (SHP/NR0B2) and fibroblast growth factor 19 (FGF‐19) which suppress the transcription of the rate‐limiting anabolic bile acid enzyme, CYP7A1 (Goodwin et al. 2000; Song et al. 2009; Russell and Setchell 1992). Hepatic clearance of bile acids is controlled by FXR. Bile salt export pump (BSEP/ABCB11), the major bile acid efflux transporter on the canalicular membrane of hepatocytes, is critical for formation of bile acid‐dependent bile flow (Strautnieks et al. 1998b). Human BSEP transcription is directly induced by FXR (Ananthanarayanan et al. 2001). Insufficient expression or nonfunctional BSEP causes cholestasis. (Strautnieks et al. 1998a; Jansen et al. 1999; Alissa et al. 2008; Davit‐Spraul et al. 2009; Whitington et al. 1994). Transporters, MRP2, BCRP, and P‐gp, also efflux bile acids into bile cannaluculi (Dawson et al. 2009). Transport of bile acids from hepatocytes into systemic circulation is mediated by basolateral efflux transporters including MRP3 and MRP4 (Rius et al. 2003; Dawson et al. 2009) and OST_*α*_ and OST_*β*_ (Landrier et al. 2006). FXR activation up‐regulates transcription of OST_*α*_ and OST_*β*_ (Boyer et al. 2006; Frankenberg et al. 2006; Landrier et al. 2006).

Primary biliary cholangitis (PBC) and primary sclerosing cholangitis (PSC) are chronic, cholestatic, and inflammatory autoimmune liver diseases (Beuers et al. 2015; Lindor et al. 2009; Sarkar and Bowlus 2016). Progressive destruction of bile ducts in PBC and PSC results in bile acid elevation in the liver and the circulation. PBC and PSC patients develop liver cirrhosis and failure eventually requiring liver transplantation; otherwise the diseases are fatal. Studies have demonstrated adaptive and compensatory mechanisms in PBC and PSC patient's livers in response to bile acids overload. Liver transporters including uptake and efflux transporters, and bile acid synthesis enzymes are adaptively changed to reduce accumulation of bile acids in hepatocytes. These compensatory mechanisms are largely regulated by the FXR (Takeyama and Sakisaka 2012).

FXR is a pharmacologically attractive target for the treatment of cholestasis in PBC, PSC, and other cholestatic diseases. Chenodeoxycholic acid (CDCA), is the most potent endogenous FXR activator (Makishima et al. 1999; Parks et al. 1999; Liu et al. 2014). Obeticholic acid (OCA), a semi‐synthetic analog of CDCA is approximately 100‐fold more potent than CDCA (Pellicciari et al. 2002). OCA was protective in a rat cholestasis model induced by estrogen (Fiorucci et al. 2005). In this model, OCA increased the bile flow and decreased the bile acid synthesis (Fiorucci et al. 2005).

Since OCA, a potent FXR agonist, is approved for the treatment of PBC, understanding its mechanistic action on genes involved in bile acid transport and synthesis is relevant. This was accomplished by utilizing sandwich‐cultured human hepatocytes (SCHH). This in vitro technique preserves the *in vivo*‐like bile acid biosynthesis and regulatory pathways (Jackson et al. 2016) including uptake and efflux transporters proper localization (Hoffmaster et al. 2004; Li et al. 2009). SCHH are also capable of determining hepatobiliary distribution of endogenous bile acids (Swift et al. 2010). FXR‐regulated gene expression, transporter function, and endogenous bile acid levels were evaluated after OCA treatment in comparison to CDCA.

## Materials and Methods

CDCA, tamoxifen, and aflatoxin B were purchased from Sigma Aldrich (St. Louis, MO). OCA and its conjugates (taurine and glycine) were provided by Intercept Pharmaceuticals, Inc. (San Diego, CA). Primary human hepatocyte cultures were seeded and maintained utilizing propriety cell culture media formulations developed at Qualyst Transporter Solutions (Durham, NC). Cell culture base medium and supplements were purchased from Gibco (Carlsbad, CA) and Corning (Tewksbury, MA). CellTiter‐Glo^®^ Luminescent Cell Viability Assays were purchased from Promega (Madison, WI). All quantitative real‐time polymerase chain reaction (qRT‐PCR) reagents were purchased from Life Technologies (Carlsbad, CA). Pierce™ BCA™ Protein Assays were purchased from Thermo Fisher Scientific (Waltham, MA).

### Sandwich‐cultured human hepatocyte culture and treatment

SCHH were prepared by Qualyst Transporter Solutions using cryopreserved human hepatocytes purchased from Triangle Research Laboratories (RTP, NC) and Xenotech (Lenexa, KS). Hepatocytes were QTS Transporter Certified™. QTS Certification signifies that SCHH reestablishes a functional bile canalicular network capable of supporting hepatic drug uptake and biliary efflux function. Transporter Certified™ cryopreserved hepatocytes in sandwich culture are the optimal system for evaluating hepatic transporter interaction potential of new chemical entities. The preparation and treatment of SCHH are described in the Appendix, Section 1.1.1.

### Cytotoxicity assessment

#### Morphological assessment

SCHH were treated with CDCA, OCA, glyco‐OCA, and tauro‐OCA (0.1, 0.316, 1.0, 3.16, 10, 31.6, 100 *μ*mol/L) or cytotoxicity‐positive controls (50 *μ*mol/L tamoxifen, 10 *μ*mol/L aflatoxin) for 72 h with daily medium change. Cell morphology was evaluated at 24, 48, and 72 h using phase contrast microscopy for each of the treatment groups on a daily basis. Images were captured using a Zeiss Axiovert 40CFL microscope equipped with phase contrast optics, AxioCam MRc camera, and AxioVision imaging software (V4.6.1).

Morphology of the hepatocyte cultures was compared to controls for any morphological alterations (e.g., changes in cell shape, cytoplasmic alterations, accumulation of vacuoles suggestive of dilated organelles and lipid droplets) indicative of cytotoxicity (Guillouzo et al. 1997; Tyson and Green 1987).

#### Biochemical evaluation of cell viability

Cell viability of hepatocytes was assessed by determining the amount of ATP present after 72 h of exposure to OCA, CDCA, glyco‐OCA tauro‐OCA, and the positive controls tamoxifen and aflatoxin B using CellTiter‐Glo Luminescent Cell Viability Assays from Promega (Madison, WI) following manufacturer's procedures. Each test condition was performed in triplicate.

### Total RNA isolation and qRT‐PCR

Following 72 h of treatment with CDCA (0.1, 0.316, 1.0, 3.16, 10, 31.6, 100 *μ*mol/L), or OCA, glyco‐OCA, or tauro‐OCA (0.00316, 0.01, 0.0316, 0.1, 0.316, 1.0, 3.16 *μ*mol/L), SCHH were washed and lysed for total RNA isolation using Qiagen RNeasy kit following manufacturer's instructions. Details are described in Appendix Section 1.2.1

### Bile acid profiling and hepatobiliary disposition assessment

Following 72 h of treatment with CDCA (100 *μ*mol/L) or OCA (1.0 *μ*mol/L), the endogenous bile acid composition and hepatobiliary disposition of d_8_‐TCA in SCHH compartments (cell, bile pocket, and cell culture medium) were determined using B‐CLEAR^®^ technology. See Appendix Section 1.3.1 for detailed descriptions of cell culture preparation, experimental procedure, and calculations for biliary accumulation, biliary excretion index (BEI), and intracellular concentration (ICC) of d_8_‐TCA. Protein content was determined using Pierce BCA protein assay kit (Thermo Fisher Scientific) following manufacturer's instructions.

### Bioanalytical of bile acid profiling and disposition assessment

Analytes (d_8_‐TCA, CA, tauro‐CA, glyco‐CA, CDCA, tauro‐CDCA, and glyco‐CDCA) were extracted from study samples (cell culture medium and hepatocyte lysates). Extraction procedure are detailed in Appendix Section 1.4.1. Prepared samples were filtered and analyzed by LC‐MS/MS using a Shimadzu binary HPLC system (Columbia, MD) and tandem mass spectrometry using Thermo Electron TSQ^®^ Quantum Discovery MAX™ (Waltham, MA) with an Ion Max ESI source operating in negative ion electrospray ionization mode using multiple reaction monitoring.

### Data analysis

The assays in this study were conducted in SCHH prepared from three individual donors except for the cytotoxicity assay that was conducted in SCHH of one liver donor. Every measurement was performed in triplicate per donor. Data were normalized to the vehicle controls (DMSO) and represent the mean ± SD from three donors. The cytotoxicity data represent means from triplicate wells from one donor. The data were analyzed with GraphPad Prism 6.0 (La Jolla, CA).

## Results

### No Cytotoxicity Induced by OCA

**Figure 1:**
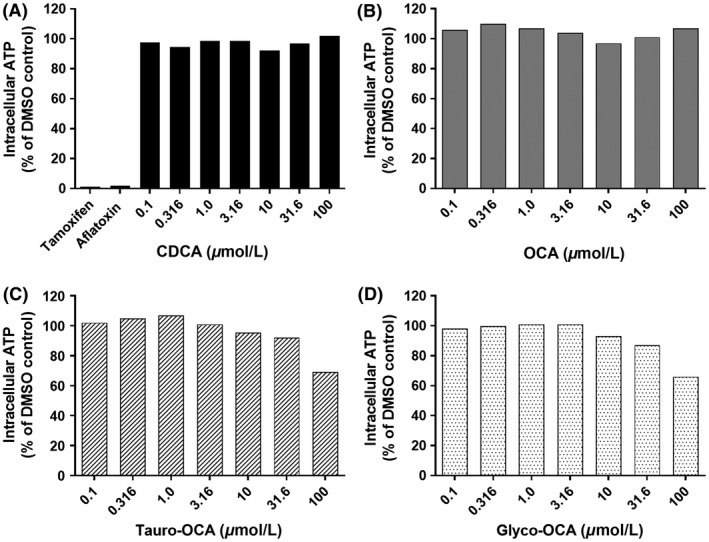
Evaluation of cell viability. ATP levels were measured in sandwich‐cultured human hepatocytes following exposure to increasing concentrations (0.1, 0.316, 1.0, 3.16, 10, 31.6, 100 *μ*mol/L) of CDCA (A), OCA (B), tauro‐OCA (C), glyco‐OCA (D), and positive controls (Tamoxifen 50 *μ*mol/L and Aflatoxin 10 *μ*mol/L) for 72 h. The data represent means from triplicate wells from 1 donor.

Compared to the vehicle control treatment, no morphological changes including loss of cuboidal cell shape, loss of cell‐to‐cell contact, and cell detachment were observed in SCHH after 72 h of exposure to OCA or CDCA (0.1, 0.316, 1.0, 3.16, 10, 31.6, 100 *μ*mol/L) (Appendix Fig. 1.3.1). Consistently, ATP depletion studies demonstrated no meaningful reduction in ATP cellular content in hepatocytes exposed to 0.1‐100 *μ*mol/L OCA or CDCA for 72 h (Fig. 1). These data suggested that OCA or CDCA were not cytotoxic up to 100 *μ*mol/L.

No marked morphological changes were observed after 72 h of treatment with 0.1‐31.6 *μ*mol/L glyco‐OCA and tauro‐OCA (Appendix Fig. 1.3.1.) indicating that glyco‐OCA or tauro‐OCA at concentrations from 0.1 to 31.6 *μ*mol/L were not cytotoxic to hepatocytes. Marked morphological changes were observed in SCHH exposed to 100 *μ*mol/L glyco‐OCA or 100 *μ*mol/L tauro‐OCA for 72 h (Appendix Fig. 1.3.1.). Cell morphology data were supported by ATP depletion studies that demonstrated ATP content in SCHH was reduced to 66.1% and 69.3% relative to control following exposure to 100 *μ*mol/L glyco‐OCA and 100 *μ*mol/L tauro‐OCA, respectively (Fig. 1). These changes indicated that glyco‐OCA and tauro‐OCA at 100 *μ*mol/L were toxic to hepatocytes.

The positive control toxicants, tamoxifen (Appendix Fig. 1.3.1) and aflatoxin B (data not shown) caused marked changes in cell morphology (e.g., loss of cuboidal cell shape and loss of cell‐to‐cell contact). The severity of morphological changes increased over time after 24, 48, and 72 h of exposure. ATP cellular content was reduced to 1.3% and 2.1% relative to control after 72 h exposure to tamoxifen and aflatoxin, respectively (Fig. 1).

### OCA activates FXR‐mediated bile acid homeostasis feedback mechanism

Using B‐CLEAR^®^ technology, the effect of 1 *μ*mol/L OCA exposure for 72 h was evaluated on total endogenous bile acid content, disposition, and bile acid synthesis in the hepatocyte, bile, and cell culture medium (CCM) from three donors in SCHH.

**Figure 2:**
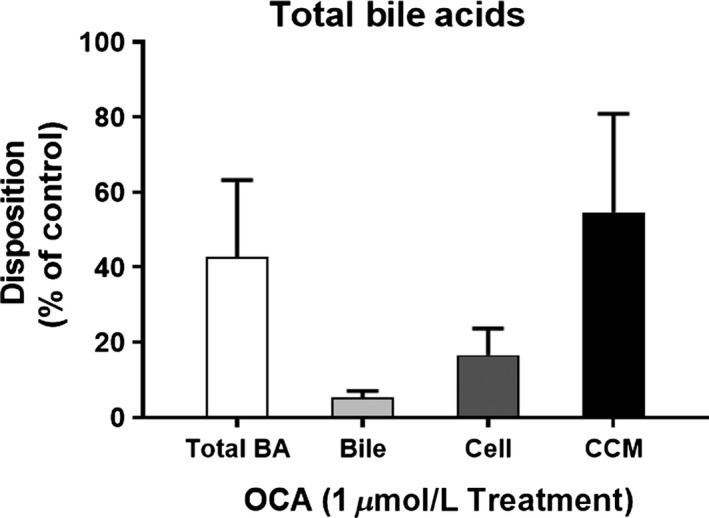
Determination of endogenous bile acid pool and disposition. Total endogenous bile acid mass was calculated including cholic acid (CA), tauro‐CA, glyco‐CA, chenodeoxycholate (CDCA), tauro‐CDCA, and glyco‐CDCA in hepatocytes (cell), bile pockets (bile), and cell culture media (CCM) following 72 h of 1 *μ*mol/L OCA. Disposition of endogenous individual bile acid components were measured and calculated in cell, CCM, and bile. The assays were conducted in SCHH from three donors and in triplicate per donor. Data were normalized to controls and represent the mean ± SD from three donors.

Total endogenous bile acid content comprised the sum of CA, glyco‐CA, tauro‐CA, CDCA, glyco‐CDCA, and tauro‐CDCA. OCA decreased the total bile acid content to 42.7 ± 20.5%, relative to control (Fig. 2). OCA correspondingly decreased total endogenous bile acid content in cell, bile, and CCM to 16.6 ± 7.2%, 5.4 ± 1.7%, and 54.6 ± 26.3%, respectively, relative to the control.

The expression of genes involved in bile acid synthesis including SHP, FGF‐19, CYP7A1, Cytochrome P450 Family 7 Subfamily B Member 1 (CYP7B1), Cytochrome P450 Family 8 Subfamily B Member 1 (CYP8B1), Bile Acid‐CoA:Amino Acid N‐Acyltransferase (BAAT), and bile acid acyl‐CoA synthetase (BACS), was evaluated in SCHH from three donors following 72 h exposure to increasing concentrations of OCA (0.00316–3.16 *μ*mol/L) or CDCA (0.1‐100 *μ*mol/L).

**Figure 3:**
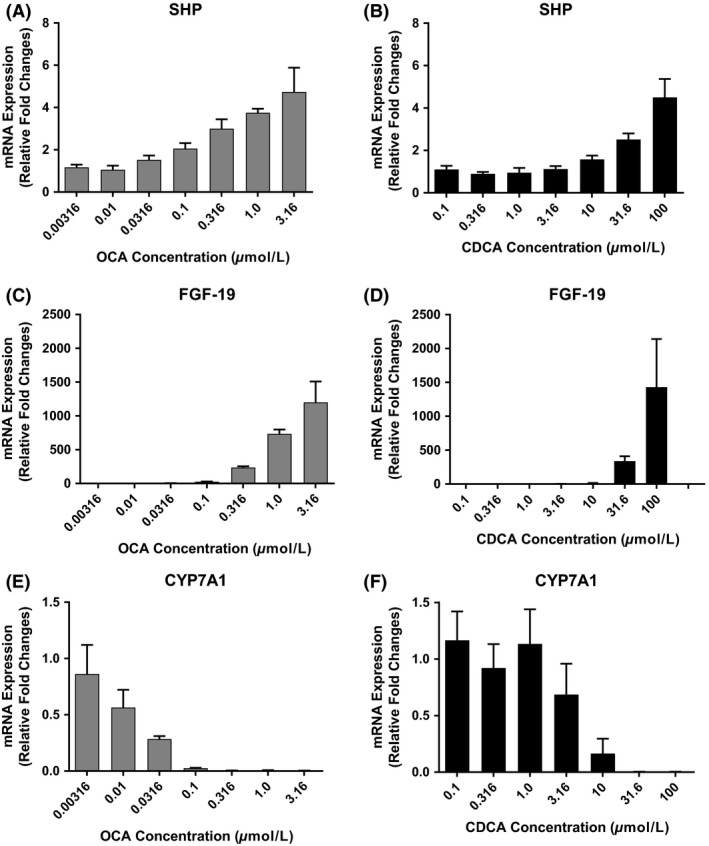
Measurement of mRNA expression of genes involved in bile acid synthesis and metabolism in Human Primary Hepatocytes. Sandwich‐cultured human hepatocytes from three donors were treated for 72 h with CDCA (0.1, 0.316, 1.0, 3.16, 10, 31.6, 100 *μ*mol/L) or OCA (0.00316, 0.01, 0.0316, 0.1, 0.316, 1.0, 3.16 *μ*mol/L). SHP (A, B), FGF‐19 (C, D), CYP7A1 (E, F), were evaluated following 72 h of exposure to increasing concentrations of CDCA and OCA using gene‐specific TaqMan^®^ assays. PCR reactions were normalized to control. The data represent means ± SD from three donors. Statistical data are presented in Appendix, Figure 1.3.2 and Appendix, Table 1.2.1

Figure 3 illustrates the effect of OCA and CDCA on the mRNA levels of SHP, FGF‐19, and CYP7A1. SHP and FGF‐19 are modulators of CYP7A1 activity. CYP7A1 is the rate‐limiting enzyme of bile acid synthesis. As OCA and CDCA cell culture concentration were increased, the genes encoding SHP and FGF‐19 mRNA levels increased; as postulated, CYP7A1 mRNA deceased. Specifically, OCA at 1 *μ*mol/L increased SHP mRNA to 3.7 ± 0.2‐fold and FGF‐19 mRNA to 735 ± 63‐fold above vehicle control. Similarly, CDCA at 100 *μ*mol/L increased SHP and FGF‐19 mRNA levels to 4.5 ± 0.9‐fold and 1430 ± 712‐fold, respectively, above control. Correspondingly, increased concentration of OCA and CDCA reduced the expression of CYP7A1 by 99%.

Dose proportionality determinations corroborate the effect of OCA and CDCA on SHP, FGF‐19, and CYP7A1 (Appendix Fig. 1.3.2; Appendix Table 1.2.1). Slopes were considered dose linear if the 0.95 CI did not cross through zero and contained the slope value. Meeting these requirements, a slope of 1 is dose proportional; a slope less than 1 is linear but less than dose proportional; and a slope greater than 1 is linear but more than dose proportional. With increasing doses of OCA, significant positive slopes (0.95 CI) of 0.2641 (0.2235–0.2993) and 1.333 (1.191–1.474), were determined for SHP and FGF‐19, respectively. Similar trends were observed for CDCA in which SHP and FGF‐19 slopes were 0.4003 (0.3295–0.4711) and 2.039 (1.683–2.395), respectively. In contrast, increased doses of OCA and CDCA decreased the production of CYP7A1 mRNA in a dose proportional manner; −1.129 (−1.348 to −0.9095) and −2.48 (−3.499 to −1.462), respectively. In addition, correlation plots were constructed between SHP and CYP7A1 mRNA levels as OCA or CDCA dose increased. It is understood that as SHP expression rises, there is a concomitant suppression of CYP7A1. Correlation statistics confirmed this assumption; significant correlations (*R* ^2^) between SHP and CYP7A1 were 0.8491 and 0.7708 for OCA and CDCA treatment, respectively (Appendix Fig. 1.3.3; Appendix Table 1.2.2.).

No marked changes were observed in CYP7B1, CYP8B1, BAAT, and BACS mRNA (Appendix Fig. 1.3.4.).

### Hepatobiliary disposition of d_8_‐TCA

**Figure 4:**
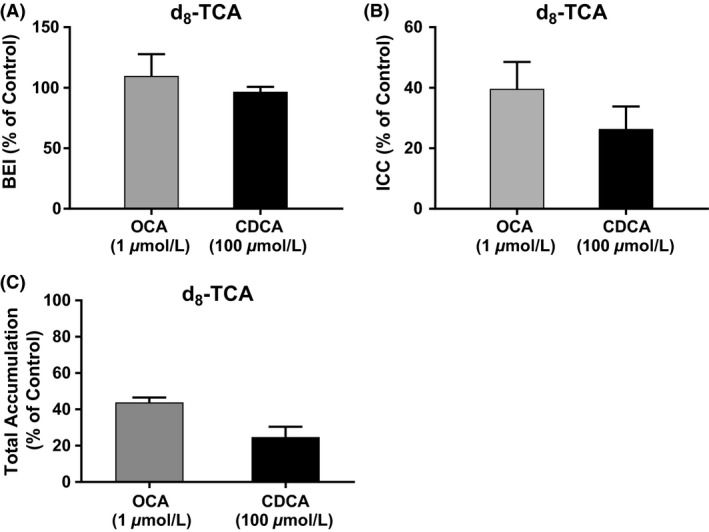
Assessment of the hepatobiliary disposition of bile acids utilizing B‐CLEAR ^®^ technology. Sandwich‐cultured human hepatocytes from three donors were treated with CDCA (100 *μ*mol/L) and OCA (1 *μ*mol/L) for 72 h. A probe bile acid, d_8_‐TCA (2.5 *μ*mol/L), was incubated in sandwich‐cultured human hepatocytes for 30 min in Ca^++^ Plus (+) buffer and Ca^++^ Minus (‐) buffer. Total accumulation of d_8_‐TCA levels (hepatocyte + bile) was measured in Ca^++^ Plus (+) buffer and intracellular levels in Ca^++^ Minus (−) buffer. The calculated BEI (A), intracellular concentration (ICC, (B)) and total accumulation (C) were normalized to controls and represent the mean ±  SD from three donors.

Using B‐CLEAR^®^ technology, the hepatobiliary disposition of d_8_‐TCA, a model bile acid, was determined in the hepatocyte and bile following 72 h exposure to 1 *μ*mol/L OCA or 100 *μ*mol/L CDCA. Compared with control, the BEI of d_8_‐TCA in SCHH was unchanged after 72 hour exposure to OCA or CDCA (109.8 ± 18.0% and 96.6 ± 4.1% relative to control, respectively, Fig. 4A). However, exposure to OCA or CDCA decreased d_8_‐TCA hepatic intracellular concentrations ICC to 39.6 ± 8.9% and 26.4 ± 7.5%, respectively, relative to control (Fig. 4B; Appendix Section 1.3.1. for calculations). Total disposition of d_8_‐TCA was reduced to 43.8 ± 2.8% and 24.7 ± 5.7%, relative to control following OCA or CDCA exposure, respectively (Fig. 4C).

### OCA increases bile acid efflux transporter gene expression

The gene expression of uptake transporters on the hepatocyte basolateral membrane (sodium‐taurocholate cotransporting polypeptide [NTCP]; organic anion transporting polypeptides 1B1, 1B3, 2B1 [OATP1B1, OATP1B3, and OATP2B1]); hepatocyte basolateral efflux transporters (multidrug resistance‐associated protein 3 and 4 [MRP3 and MRP4], OST_*α*_, OST_*β*_; canalicular efflux transporters (multidrug resistance‐associated protein 2 [MRP2], breast cancer resistance protein [BCRP], P‐glycoprotein [P‐gp], and BSEP), were determined following 72 h of exposure to increasing concentrations of OCA or CDCA).

**Figure 5:**
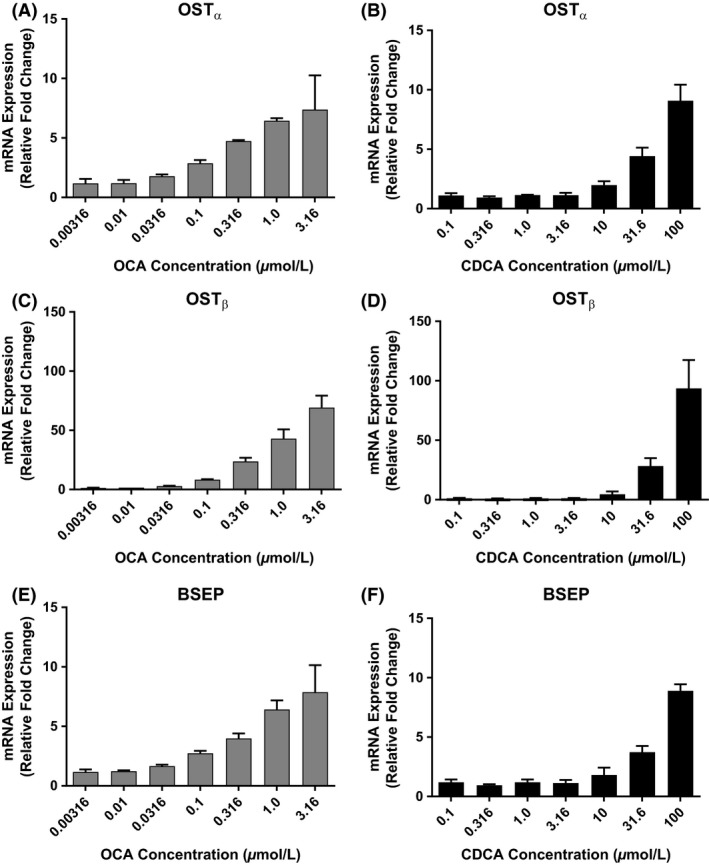
Measurement of mRNA Expression of Bile Acid Efflux Transporters (OST_*α*_, OST_*β*_, and BSEP). After Treatment with OCA or CDCA in Human Primary Hepatocytes. Sandwich‐cultured human hepatocytes from three donors were treated for 72 h with CDCA (0.1, 0.316 1.0, 3.16, 10, 31.6, 100 *μ*mol/L) or OCA (0.00316, 0.01, 0.0316, 0.1, 0.316, 1.0, 3.16 *μ*mol/L). OST_*α*_ (A, B), and OST_*β*_ (C, D), and bile acid transporter, BSEP (E, F), were evaluated using gene‐specific TaqMan^®^ assays. PCR reactions were performed in triplicate wells for each donor and normalized to control. The data represent mean ± SD from three donors.

Exposure to OCA at 1 *μ*mol/L increased expression of basolateral efflux heterodimers OST_*α*_ mRNA and OST_*β*_ mRNA by 6.4 ± 0.2‐fold and 42.9 ± 7.9‐fold, respectively, relative to control (Fig. 5A and C). Similarly, increases in OST_*α*_ and OST_*β*_ expression were observed following CDCA exposure at the highest dose (100 *μ*mol/L) [9.1 ± 1.3‐fold and 93.6 ± 23.8‐fold relative to control, respectively (Fig. 5B and D)]. Expression of the canalicular efflux transporter, BSEP, was 6.4 ± 0.8‐fold greater than the vehicle control following exposure to 1 *μ*mol/L OCA (Fig. 5E). Likewise, exposure to 100 *μ*mol/L CDCA increased the expression of BSEP mRNA to 8.9 ± 0.6‐fold above control (Fig. 5F). These data were corroborated using slope determinations for OCA and CDCA with respect to OST_*α*_, OST_*β*_, and BSEP mRNA expression (Appendix Fig. 1.3.5 and Appendix Table 1.2.3.). In each treatment, as analyte concentration increased, there was a corresponding incremental increase in mRNA levels. Dose–response slopes were linear but less than dose proportional with the exception of the dose proportional slope for CDCA‐OST_*β*_ curve.

No marked changes were observed in the expression of basolateral bile acid uptake transporters NTCP, OATP1B1, OATP1B3, and OATP2B1 in SCHH following 72 h exposure to OCA or CDCA. These results suggest that uptake may not contribute to the decrease observed in total bile acid accumulation or bile acid ICC (e.g. d_8_‐TCA). Similarly, the expression of other efflux transporters, P‐gp, MRP2, MRP3, MRP4, and BCRP, were unchanged in SCHH following treatment with either OCA or CDCA (Appendix Fig. 1.3.6.).

Using binding assays, glyco‐OCA and tauro‐OCA have been shown to be equipotent agonist compared to parent at FXR. (data not shown). Confirmation of similar agonist effect of glyco‐OCA and tauro‐OCA at FXR was demonstrated using a pharmacological platform measuring mRNA levels for CYP7A1, SHP, FGF‐19, BSEP, OST_*α*_, OST_*β*_, other CYP enzymes, and transporters (Appendix Fig. 1.3.7.). For each parameter examined, the conjugated metabolites produced the same level of mRNA expression as OCA.

## Discussion

**Figure 6:**
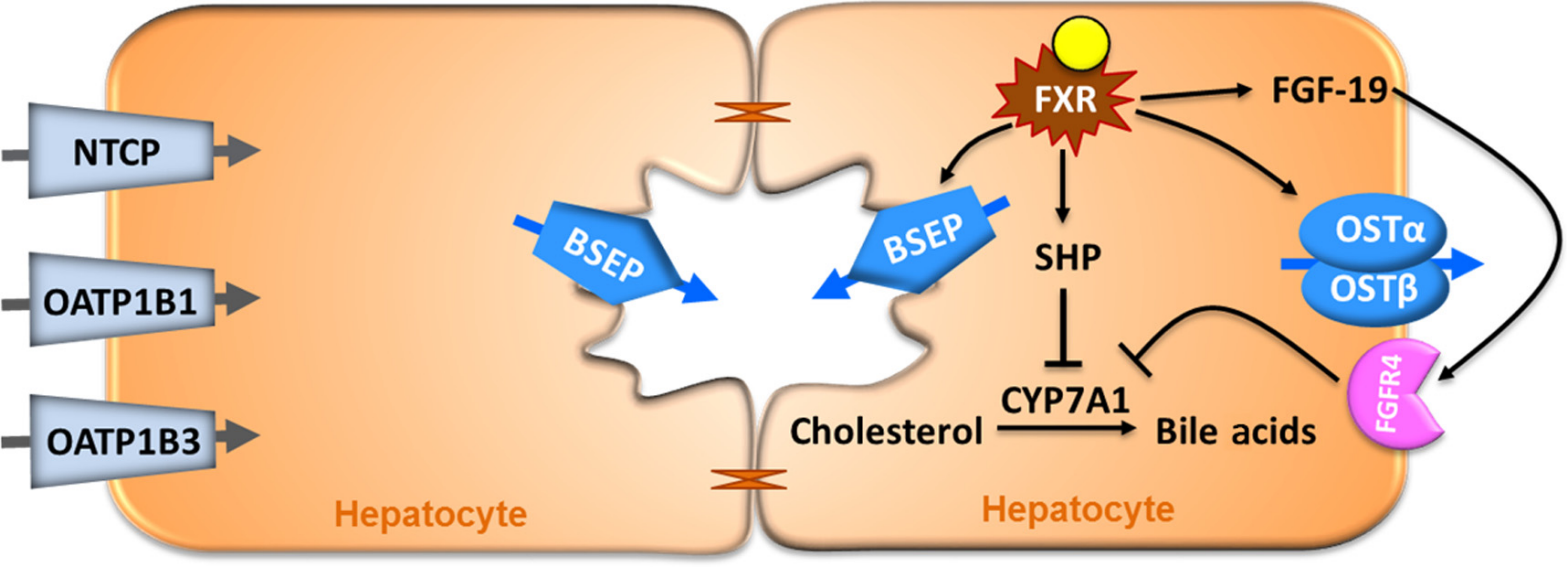
Mechanisms of bile acid homeostasis. Activation of FXR by CDCA or OCA results in increased expression of small heterodimer partner (SHP) and fibroblast growth factor 19 (FGF‐19), genes that suppress Cholesterol 7*α*‐hydroxylase (CYP7A1), the rate‐limiting enzyme in the de novo bile acid synthesis pathway. FXR also directly regulates bile acids via induction of the bile acid salt export pump (BSEP), which effluxes bile acids from hepatocytes to bile and heterodimer organic solute transporters, OST_*α*_ and OST_*β*_, which transport bile acids form hepatocytes to blood circulation.

FXR acts as a master regulator of bile acid homeostasis (Makishima et al. 1999; Chiang 2009) as illustrated in Figure 6. Identification of FXR as a therapeutic target for the treatment of chronic liver diseases (e.g., PBC) has led to the development of more potent FXR agonists including OCA (Ali et al. 2015). OCA has higher selectivity and is approximately 100‐fold more potent on FXR than CDCA, the endogenous ligand. Similar to endogenous bile acids, OCA is metabolized to glycine and taurine conjugates, glyco‐OCA and tauro‐OCA, respectively. It has also been determined that the OCA conjugates have nearly identical activity on FXR as does OCA. To better understand the mechanism‐of‐action of OCA in humans, a validated in vitro human hepatocyte sandwich‐cultured (SCHH) model was employed to investigate gene regulation of bile acid synthesis and bile acid elimination. In addition, the OCA hepatic effects were compared to the natural occurring bile salt ligand CDCA.

Foremost, OCA and CDCA bile acids at concentrations up to 100 *μ*mol/L for 72 h did not damage sandwich‐cultured hepatocytes as evidenced by lack of cellular morphological alterations or cellular reduction in ATP (Appendix Figure 1.3.1.). Compared to conventional cultured primary hepatocytes, where OCA concentrations <3 *μ*mol/L produced cytotoxicity (data not shown). The OCA conjugates were not cytotoxic up to 31.6 *μ*mol/L; they did, however, show clear morphological changes and ATP cellular loss at 100 *μ*mol/L. Positive control toxicants damaged hepatocytes in a time‐dependent manner adding to the confidence that OCA and metabolites were not toxic at expected therapeutic concentrations. The absence of OCA cellular toxicity may likely be due to polarization of SCH hepatocytes resulting in the appropriate localization and function of bile acid transporters (Li et al. 2009; Swift et al. 2010), thus allowing natural bile acids and bile acid analogues to efflux into bile pockets and reduce intracellular accumulation and cytotoxicities (Jackson et al. 2016).

OCA at 1 *μ*mol/L for 72 h decreased total endogenous bile acid content (CA, glyco‐CA, tauro‐CA, CDCA, glyco‐CDCA, and tauro‐CDCA) by approximately 57%. Sampling separate compartments of the model, reductions in total bile acid were observed in hepatocytes, bile, and CCM (Fig. 2). In a separate experiment, total disposition of d_8_‐TCA (a prototypical bile acid) was reduced to 43.8 ± 2.8% and 24.7 ± 5.7%, relative to control following OCA or CDCA exposure, respectively (Fig. 4C). These data support the hypothesis that OCA and CDCA downregulate bile acid production in human hepatocytes. As discussed below, further work using gene biomarkers confirmed this theory.

There are numerous, complex biological cascades triggered by OCA and CDCA that regulate bile acid homeostasis. These include but are not limited to bile acid synthesis and bile acid uptake and efflux transporters. Expression of relevant genes involved in bile acid synthesis stimulated by OCA and CDCA include SHP, FGF‐19, and CYP7A1. OCA and CDCA function as FXR agonists resulting in the inhibition of bile acid synthesis. Stimulation of FXR leads to increased levels of SHP and FGF‐19. These in turn suppress the production of CYP7A1, the rate‐limiting enzyme of bile acid synthesis thereby reducing bile acid levels. Addition of either agonist to the SCHH plates increased SHP and FGF‐19 mRNA. OCA at 1 *μ*mol/L, increased mRNA levels approximately 4‐ and 735‐fold, SHP and FGF‐19, respectively, above vehicle control (Fig. 3). CDCA concentrations of 100 *μ*mol/L achieved similar effects as OCA. Concentration of OCA and CDCA at 1 and 100 *μ*mol/L, respectively, reduced expression of CYP7A1 by 99%. Dose–response analysis determined that SHP, FGF‐19, and CYP7A1 mRNA levels increased in a linear fashion with changing dose (Appendix Fig. 1.3.2). In addition, correlation plots of SHP versus CYP7A1 mRNA (Appendix Fig. 1.3.3) as OCA or CDCA dose increased, showed good correlations (*R* ^2^) in which an increase in SHP led to a decrease in CYP7A1 mRNA levels after exposure to OCA (0.849) or CDCA (0.771). These data confirm that FXR activation by OCA or CDCA results in predictable pharmacological regulation of bile acid homeostasis.

The comparison of OCA with CDCA confirmed that OCA potency on FXR is approximately 100‐fold greater than CDCA as shown by the dose‐dependent increase in the expression of respective FXR target genes, SHP and FGF‐19, and subsequent inhibition of CYP7A1 (Lee et al. 2000; Holt et al. 2003; del Castillo‐Olivares et al. 2004). De novo bile acid synthesis was substantially suppressed in OCA‐treated hepatocytes; this result is in line with reduced circulating C4 levels, a marker of bile acid synthesis in clinical studies (Hirschfield et al. 2015). OCA and CDCA had minor effects on other gene targets that include CYP7B1, CYP8B1, BAAT, and BACS.

Bile acid levels in hepatocytes and bile canaliculae are also controlled by membrane transporters. OCA (1 *μ*mol/L) and CDCA (100 *μ*mol/L) increased the expression of hepatocyte basolateral efflux transporters, OST_*α*_ mRNA (~6‐ and 9‐fold) and OST_*β*_ mRNA (~43‐ and 93‐fold); respectively. At the same agonist concentrations, expression of the canalicular efflux transporter on the apical hepatocyte membrane, BSEP, was 6‐and 9‐fold higher, OCA and CDCA, respectively, than vehicle control. In combination, OCA modulates the efflux of bile acid from the hepatocyte via the basolateral membrane transports, OST_*α*_ and OST_*β*_, and the apical membrane transporter BSEP. OST_*β*_ upregulation was more sensitive to OCA drug stimulus; approximately 8‐fold higher compared to OST_*α*_ or BSEP.

Although there was an increase in the apical efflux transporter BSEP, no apparent change in the BEI was observed. These data suggest that the increase in the basolateral efflux is much greater than the increase in the apical efflux, consistent with results for CDCA treated SCHH (Jackson et al. 2016). A follow‐up study of mechanistic modeling and simulation will be to evaluate the extent of BSEP‐mediated apical efflux, and OST_*α*_/OST_*β*_‐mediated basolateral efflux after OCA treated SCHH.

In conclusion, OCA and its glycine and taurine conjugates are selective and potent FXR agonists that reduce the total bile acid pool and intracellular concentration of potentially cytotoxic bile acids in hepatocytes. Thus, FXR activation is an important compensatory mechanism to prevent cholestatic hepatotoxicity. These results support the use of OCA to treat bile acid‐induced toxicity observed in cholestatic diseases including PBC.

## Disclosures

Yuanyuan Zhang and Jeffrey E. Edwards are employees and stock shareholders of Intercept Pharmaceuticals, Inc; Jonathan P. Jackson, Robert L. St. Claire III, Kimberly Freeman and Kenneth R. Brouwer have no conflict of interest.

## Supporting information


**Appendix S1.** Materials and Methods, tables and figures.

## References

[prp2329-bib-0001] Ali AH , Carey EJ , Lindor KD (2015). Recent advances in the development of farnesoid X receptor agonists. Ann Transl Med 3: 5. 25705637 10.3978/j.issn.2305-5839.2014.12.06 PMC4293481

[prp2329-bib-0002] Alissa FT , Jaffe R , Shneider BL (2008). Update on progressive familial intrahepatic cholestasis. J Pediatr Gastroenterol Nutr 46: 241–252. 18376240 10.1097/MPG.0b013e3181596060

[prp2329-bib-0003] Ananthanarayanan M , Balasubramanian N , Makishima M , Mangelsdorf DJ , Suchy FJ (2001). Human bile salt export pump promoter is transactivated by the farnesoid X receptor/bile acid receptor. J Biol Chemist 276: 28857–28865. 10.1074/jbc.M011610200 11387316

[prp2329-bib-0004] Beuers U , Gershwin ME , Gish RG , et al. (2015). Changing nomenclature for PBC: from ‘cirrhosis’ to ‘cholangitis’. Gastroenterology 149: 1627–1629. 26385706 10.1053/j.gastro.2015.08.031

[prp2329-bib-0005] Boyer JL , Trauner M , Mennone A , et al. (2006). Upregulation of a basolateral FXR‐dependent bile acid efflux transporter OSTalpha‐OSTbeta in cholestasis in humans and rodents. Am J Phys Gastrointest Liver Physiol 290: G1124–G1130. 10.1152/ajpgi.00539.2005 16423920

[prp2329-bib-0006] del Castillo‐Olivares A , Campos JA , Pandak WM , et al. (2004). The role of alpha1‐fetoprotein transcription factor/LRH‐1 in bile acid biosynthesis: a known nuclear receptor activator that can act as a suppressor of bile acid biosynthesis. J Biol Chemist 279: 16813–16821. 10.1074/jbc.M400646200 14766742

[prp2329-bib-0007] Chandra P , Brouwer KL (2004). The complexities of hepatic drug transport: current knowledge and emerging concepts. Pharm Res 21: 719–735. 15180326 10.1023/b:pham.0000026420.79421.8f

[prp2329-bib-0008] Chiang JY (2009). Hepatocyte nuclear factor 4alpha regulation of bile acid and drug metabolism. Expert Opin Drug Metab Toxicol 5: 137–147. 19239393 10.1517/17425250802707342 PMC3645476

[prp2329-bib-0009] Davit‐Spraul A , Gonzales E , Baussan C , et al. (2009). Progressive familial intrahepatic cholestasis. Orphanet J Rare Dis 4: 1. 19133130 10.1186/1750-1172-4-1 PMC2647530

[prp2329-bib-0010] Dawson PA , Lan T , Rao A (2009). Bile acid transporters. J Lipid Res 50: 2340–2357. 19498215 10.1194/jlr.R900012-JLR200 PMC2781307

[prp2329-bib-0011] Fiorucci S , Clerici C , Antonelli E , et al. (2005). Protective effects of 6‐ethyl chenodeoxycholic acid, a farnesoid X receptor ligand, in estrogen‐induced cholestasis. J Pharmacol Exp Ther 313: 604–612. 15644430 10.1124/jpet.104.079665

[prp2329-bib-0012] Forman BM , Goode E , Chen J , et al. (1995). Identification of a nuclear receptor that is activated by farnesol metabolites. Cell 81: 687–693. 7774010 10.1016/0092-8674(95)90530-8

[prp2329-bib-0013] Frankenberg T , Rao A , Chen F , et al. (2006). Regulation of the mouse organic solute transporter alpha‐beta, Ostalpha‐Ostbeta, by bile acids. Am J Physiol Gastrointest Liver Physiol 290: G912–G922. 16357058 10.1152/ajpgi.00479.2005

[prp2329-bib-0014] Ghibellini G , Vasist LS , Leslie EM , et al. (2007). In vitro‐in vivo correlation of hepatobiliary drug clearance in humans. Clin Pharmacol Ther 81: 406–413. 17235333 10.1038/sj.clpt.6100059

[prp2329-bib-0015] Goodwin B , Jones SA , Price RR , et al. (2000). A regulatory cascade of the nuclear receptors FXR, SHP‐1, and LRH‐1 represses bile acid biosynthesis. Mol Cell 6: 517–526. 11030332 10.1016/s1097-2765(00)00051-4

[prp2329-bib-0016] Guillouzo A , Morel F , Langouet S , et al. (1997). Use of hepatocyte cultures for the study of hepatotoxic compounds. J Hepatol 26(Suppl 2): 73–80. 9204412 10.1016/s0168-8278(97)80499-0

[prp2329-bib-0017] Hirschfield GM , Mason A , Luketic V , et al. (2015). Efficacy of obeticholic acid in patients with primary biliary cirrhosis and inadequate response to ursodeoxycholic acid. Gastroenterology 148(751–61): e8. 25500425 10.1053/j.gastro.2014.12.005

[prp2329-bib-0018] Hoffmaster KA , Turncliff RZ , LeCluyse EL , et al. (2004). P‐glycoprotein expression, localization, and function in sandwich‐cultured primary rat and human hepatocytes: relevance to the hepatobiliary disposition of a model opioid peptide. Pharmaceutical Res. 21: 1294–1302. 10.1023/b:pham.0000033018.97745.0d 15290872

[prp2329-bib-0019] Holt JA , Luo G , Billin AN , et al. (2003). Definition of a novel growth factor‐dependent signal cascade for the suppression of bile acid biosynthesis. Genes Dev 17: 1581–1591. 12815072 10.1101/gad.1083503 PMC196131

[prp2329-bib-0020] Jackson JP , Freeman KM , Friley WW , St. Claire RL III , Black C , Brouwer KR (2016). Basolateral efflux transporters: a potentially important pathway for the prevention of cholestatic hepatotoxicity. Applied In Vitro Toxicology 2: 207–216.

[prp2329-bib-0021] Jansen PL , Strautnieks SS , Jacquemin E , et al. (1999). Hepatocanalicular bile salt export pump deficiency in patients with progressive familial intrahepatic cholestasis. Gastroenterology 117: 1370–1379. 10579978 10.1016/s0016-5085(99)70287-8

[prp2329-bib-0022] Landrier JF , Eloranta JJ , Vavricka SR , et al. (2006). The nuclear receptor for bile acids, FXR, transactivates human organic solute transporter‐alpha and ‐beta genes. Am J Phys Gastrointest Liver Physiol 290: G476–G485. 10.1152/ajpgi.00430.2005 16269519

[prp2329-bib-0023] Lee YK , Dell H , Dowhan DH , et al. (2000). The orphan nuclear receptor SHP inhibits hepatocyte nuclear factor 4 and retinoid X receptor transactivation: two mechanisms for repression. Mol Cell Biol 20: 187–195. 10594021 10.1128/mcb.20.1.187-195.2000 PMC85074

[prp2329-bib-0024] Li N , Bi YA , Duignan DB , et al. (2009). Quantitative expression profile of hepatobiliary transporters in sandwich cultured rat and human hepatocytes. Mol Pharm 6: 1180–1189. 19545175 10.1021/mp900044x

[prp2329-bib-0025] Lindor KD , Gershwin ME , Poupon R , et al. (2009). Primary biliary cirrhosis. Hepatology 50: 291–308. 19554543 10.1002/hep.22906

[prp2329-bib-0026] Liu X , Chism JP , LeCluyse EL , et al. (1999). Correlation of biliary excretion in sandwich‐cultured rat hepatocytes and in vivo in rats. Drug Metab Dispos 27: 637–644. 10348791

[prp2329-bib-0027] Liu J , Lu H , Lu YF , et al. (2014). Potency of individual bile acids to regulate bile acid synthesis and transport genes in primary human hepatocyte cultures. Toxicol Sci 141: 538–546. 25055961 10.1093/toxsci/kfu151 PMC4271050

[prp2329-bib-0028] Makishima M , Okamoto AY , Repa JJ , et al. (1999). Identification of a nuclear receptor for bile acids. Science 284: 1362–1365. 10334992 10.1126/science.284.5418.1362

[prp2329-bib-0029] Parks DJ , Blanchard SG , Bledsoe RK , et al. (1999). Bile acids: natural ligands for an orphan nuclear receptor. Science 284: 1365–1368. 10334993 10.1126/science.284.5418.1365

[prp2329-bib-0030] Pellicciari R , Fiorucci S , Camaioni E , et al. (2002). 6alpha‐ethyl‐chenodeoxycholic acid (6‐ECDCA), a potent and selective FXR agonist endowed with anticholestatic activity. J Med Chem 15(45): 3569–3572. 10.1021/jm025529g 12166927

[prp2329-bib-0031] Prawitt J , Caron S , Staels B (2014). Glucose‐lowering effects of intestinal bile acid sequestration through enhancement of splanchnic glucose utilization. Trends Endocrinol Metab 25: 235–244. 24731596 10.1016/j.tem.2014.03.007

[prp2329-bib-0032] Rius M , Nies AT , Hummel‐Eisenbeiss J , et al. (2003). Cotransport of reduced glutathione with bile salts by MRP4 (ABCC4) localized to the basolateral hepatocyte membrane. Hepatology 38: 374–384. 12883481 10.1053/jhep.2003.50331

[prp2329-bib-0033] Russell DW , Setchell KD (1992). Bile acid biosynthesis. Biochemistry 31: 4737–4749. 1591235 10.1021/bi00135a001

[prp2329-bib-0034] Sarkar S , Bowlus CL (2016). Primary sclerosing cholangitis: multiple phenotypes. Multiple Approaches. Clin Liver Dis 20: 67–77. 26593291 10.1016/j.cld.2015.08.005 PMC4662051

[prp2329-bib-0035] Song KH , Li T , Owsley E , et al. (2009). Bile acids activate fibroblast growth factor 19 signaling in human hepatocytes to inhibit cholesterol 7alpha‐hydroxylase gene expression. Hepatology 49: 297–305. 19085950 10.1002/hep.22627 PMC2614454

[prp2329-bib-0036] Strautnieks SS , Bull LN , Knisely AS , et al. (1998a). A gene encoding a liver‐specific ABC transporter is mutated in progressive familial intrahepatic cholestasis. Nat Genet 20: 233–238. 9806540 10.1038/3034

[prp2329-bib-0037] Strautnieks SS , Bull LN , Knisely AS , et al. (1998b). A gene encoding a liver‐specific ABC transporter is mutated in progressive familial intrahepatic cholestasis. Nat Genet 20: 233–238. 9806540 10.1038/3034

[prp2329-bib-0038] Swift B , Pfeifer ND , Brouwer KL (2010). Sandwich‐cultured he patocytes: an in vitro model to evaluate hepatobiliary transporter‐based drug interactions and hepatotoxicity. Drug Metab Rev 42(3): 446–471. 20109035 10.3109/03602530903491881 PMC3097390

[prp2329-bib-0039] Takeyama Y , Sakisaka S (2012). Hepatobiliary membrane transporters in primary biliary cirrhosis. Hepatol Res 42: 120–130. 22175826 10.1111/j.1872-034X.2011.00912.x

[prp2329-bib-0040] Teodoro JS , Rolo AP , Palmeira CM (2011). Hepatic FXR: key regulator of whole‐body energy metabolism. Trends Endocrinol Metab 22: 458–466. 21862343 10.1016/j.tem.2011.07.002

[prp2329-bib-0041] Thomas C , Gioiello A , Noriega L , et al. (2009). TGR5‐mediated bile acid sensing controls glucose homeostasis. Cell Metab 10: 167–177. 19723493 10.1016/j.cmet.2009.08.001 PMC2739652

[prp2329-bib-0042] Tyson CA , Green CE (1987). 6‐cytotoxicity measures: choices and methods pp. 119–158. *In* Rauchman E.J., *ed* The Isolated hepatocyte: Use in Toxicology and Xenobiotic Biotransformations. Elsevier

[prp2329-bib-0043] Wang H , Chen J , Hollister K , et al. (1999). Endogenous bile acids are ligands for the nuclear receptor FXR/BAR. Mol Cell 3: 543–553. 10360171 10.1016/s1097-2765(00)80348-2

[prp2329-bib-0044] Watanabe M , Houten SM , Mataki C , et al. (2006). Bile acids induce energy expenditure by promoting intracellular thyroid hormone activation. Nature 439: 484–489. 16400329 10.1038/nature04330

[prp2329-bib-0045] Whitington PF , Freese DK , Alonso EM , et al. (1994). Clinical and biochemical findings in progressive familial intrahepatic cholestasis. J Pediatr Gastroenterol Nutr 18: 134–141. 7912266 10.1097/00005176-199402000-00003

