# Supplementary material for: Obeticholic acid, a selective farnesoid X receptor agonist, regulates bile acid homeostasis in sandwich‐cultured human hepatocytes
Source: Pharmacol Res Perspect. 2017 Jun 21;5(4):e00329. doi: 10.1002/prp2.329 (PMC5684861; doi:10.1002/prp2.329)
Supplement: Supplementary file 1 — Appendix S1. Materials and Methods, tables and figures. [file PRP2-5-e00329-s001.docx]

# Appendix

## Materials and Methods

### Sandwich-Cultured Human Hepatocyte Culture and Treatment

Cryopreserved hepatocytes were thawed following manufacturer’s thawing instructions. SCHH were prepared by plating cryopreserved hepatocytes suspended in QualGro^TM^ Seeding Medium, Qualyst Transporter Solutions propriety hepatocyte seeding medium, at a density of 0.8 x 10^6^ cells/mL onto BioCoat^®^ 24‑well cell culture plates. Following plating, cells were allowed to attach for 2-4 hours, rinsed, and fed with 37°C seeding medium. Eighteen to 24 hours later, cells were fed and over‑laid with QualGro^TM^ Hepatocyte Culture Induction Medium supplemented with 0.35 mg/mL Matrigel^®^ (Corning). Cells were maintained in QualGro^TM^ Hepatocyte Culture Induction Medium until consumed in studies and kept at 37°C in a humidified incubator with 95% air/5% CO_2_.

SCHH were treated daily for 3 consecutive days starting on day 2 of culture with OCA, CDCA, glyco-OCA, tauro-OCA, or cytotoxicity positive controls (50 µM tamoxifen, 10 µM aflatoxin) to evaluate gene expression, bile acid composition and disposition, and cytotoxicity. DMSO stock solutions were diluted directly into QualGro^TM^ Hepatocyte Culture Induction Medium. DMSO concentration in the medium was ≤1%.

### Total RNA Isolation and qRT-PCR

Pooled total RNA (500 ng) from triplicate wells was converted to cDNA following the manufacturer’s procedure for the High Capacity cDNA Archive Kit (Thermo Fisher Scientific). Human gene-specific TaqMan® primers and probes were purchased from Thermo Fisher Scientific. Glyceraldehyde-3-phosphate dehydrogenase (GAPDH) was used as a house-keeping gene. Relative-fold mRNA content was determined for each treatment group relative to the endogenous control gene expression and the calibrator, 0.1% vehicle control (DMSO) using the ViiA™ 7 system software.

### Bile Acid Profiling and Hepatobiliary Disposition Assessment

Cell culture medium was removed from the hepatocytes and stored at ‑80°C. After removal of cell culture medium, hepatocytes were washed twice with Plus (+) buffer (buffer containing Ca^++^) or Minus (-) buffer (buffer without Ca^++^). The wash solutions were removed and replaced with fresh Plus (+) or Minus (-) buffer and incubated for 10 minutes at 37°C to condition hepatocytes. After conditioning, the Plus (+) and Minus (-) buffers were removed and replaced with dosing solution composed of Plus (+) buffer with 2.5 µM d_8_‑TCA with 4% BSA. The dosing solution was incubated at 37°C for 30 minutes. Following the incubation, the dosing solutions were removed and the hepatocytes were washed three times with ice-cold Plus (+) buffer. The plates were frozen at -80°C until processed for bioanalysis to determine protein content, composition and disposition of endogenous bile acids, and disposition of d_8_-TCA.

All mass values from hepatobiliary disposition studies were normalized to the mean protein (mg) per well. Cellular accumulation determined in Minus (-) buffer (Cellular Accumulation_Minus (-) Buffer_) represents the total mass of analyte inside the hepatocyte at the end of the incubation time period ([Ghibellini and Vasist, 2007](#_ENREF_14)). Total accumulation determined in Plus (+) buffer (Accumulation_Plus (+) Buffer_) represents the total mass of analyte taken up and excreted into bile pockets (Cells+Bile) ([Ghibellini and Vasist, 2007](#_ENREF_14)). The biliary accumulation represents the total mass of analyte excreted into the bile pockets and was calculated as follows:

$Bile Accumulation=Total Accumulation_{\left( Plus \left( + \right) Buffer \right)}-Cellular Accumulation{}_{\left( Minus \left( - \right) Buffer \right)}{}$

The biliary excretion index (BEI) represents the fraction of the analyte taken up and excreted into the bile pockets and was calculated according to the calculation below ([Ghibellini and Vasist, 2007](#_ENREF_14); [Chandra and Brouwer, 2004](#_ENREF_6); [Liu and Chism, 1999](#_ENREF_27)):

$$BEI=100*\frac{Bile Accumulation}{Total Accumulation_{\left( Plus \left( + \right)Buffer \right)}}$$

The intracellular concentration (ICC) of each analyte was calculated by dividing the mass of analyte in the hepatocytes (Cellular Accumulation _Minus (-) Buffer_) by the estimated human hepatocyte intracellular fluid volume of 7.69 µL/mg protein (Qualyst internal data) as indicated below:

$$ICC=\frac{Cellular Accumulation_{\left( Minus \left( - \right)Buffer \right)}}{7.69 \mu L/mg}$$

Total accumulation and cellular accumulation values were generated from triplicate wells and are represented by the calculated mean and standard deviation of the mean (SD) from these biological replicates.

### Bioanalytical of Bile Acid Profiling and Disposition Assessment

Analytes (d8-TCA, CA, tauro-CA, glyco-CA, CDCA, tauro-CDCA, and glyco-CDCA) were extracted from study samples (cell culture medium and hepatocyte lysates). A volume of 500 µL of lysis solution, 70:30 methanol:water (v:v) containing 25 nM internal standard (d5-TCA), was added to each well. Following shaking for >15 min the cell lysate was filtered into the deep well plate by centrifugation. Analytes were extracted from cell culture medium samples using a protein precipitation procedure. Cell culture medium samples (100 µL) were mixed by shaking >15 minutes with 300 µL of methanol containing internal standard (d5-TCA) in a protein precipitation filter plate (Millipore MDRPNP4) and then filtered into a deep-well plate by centrifugation. The filtrates from cell lysate samples and cell culture medium samples were dried by evaporation under nitrogen. The dried filtrates from cell lysate were reconstituted in 100 or 120 µL of sample diluent (60:40 methanol:water containing 10 mM ammonium acetate), and the filtrates from cell culture medium samples in 150 µL volume. The reconstituted samples were transferred to a Millipore 0.45 μm filter plate (Millipore MSHVN45) and filtered into a Costar 3957 plate by centrifugation and sealed with a silicone capmat prior to LC-MS/MS analysis.

Standard and quality control samples were prepared by spiking previously frozen analytical plates with a known concentration of compounds typically ranging from 0.5 to 500 pmol/well. These lysate controls were further processed as cell lysate sample. Separate standards and quality control (QC) samples were prepared for cell culture media study samples and post incubation supernatant media samples. Standard and QC media solutions were prepared by diluting spiking solutions 1:10 in appropriate media over a concentration range (typically 0.05 – 10 μM). Further processing of media standard and QCs was completed as described in sample preparation of cell culture medium samples.

## Appendix Tables

**Table 1.2.1. Dose Linearity Statistics for Appendix Figure 1.3.2**

| Parameter | OCA | | | CDCA | | |
| --- | --- | --- | --- | --- | --- | --- |
| **Target** | **SHP** | **FGF-19** | **CYP7A1** | **SHP** | **FGF-19** | **CYP7A1** |
| **Dose Range (µM)** | 0.01-3.16 | 0.01-3.16 | 0.00316-1.0 | 3.16-100 | 3.16-100 | 3.16-31.6 |
| **Slope** | 0.2614 | 1.333 | -1.129 | 0.4003 | 2.039 | -2.48 |
| **0.95 CI** | 0.2235 to 0.2993 | 1.191 to 1.474 | -1.348 to 0.9095 | 0.3295 to 0.4711 | 1.683 to 2.395 | -3.499 to-1.462 |
| **Dose-response** | linear | linear | linear | linear | linear | linear |
| **Number of doses** | 18 | 18 | 16 | 12 | 12 | 8 |
| **Number of subjects** | 21 | 21 | 19 | 21 | 21 | 19 |

**Table 1.2.2. Correlation Statistics between SHP-mRNA and CYP7A1-mRNA in response to increasing concentrations of OCA and CDCA for Appendix Figure 3**

| Parameter | SHP-mRNA correlation with CYP7A1-mRNA | |
| --- | --- | --- |
| **Treatment** | **OCA** | **CDCA** |
| **Dose Range (µM)** | 0.00316-3.16 | 0.1-100 |
| **Correlation coefficient (R)** | -0.9215 | -0.8779 |
| **0.95 CI** | -0.9698 to -0.8036 | -0.9524 to -0.7048 |
| **P-value (two-tailed)** | <0.0001 | <0.0001 |
| **R squared** | 0.8491 | 0.7708 |
| **Number of XY Pairs** | 19 | 19 |

**Table 1.2.3.** **Dose Linearity Statistics for Appendix Figure 5**

| Parameter | OCA | | | CDCA | | |
| --- | --- | --- | --- | --- | --- | --- |
| **Target Response** | **OST_α_** | **OST_β_** | **BSEP** | **OST_α_** | **OST_β_** | **BSEP** |
| **Dose Range (µM)** | 0.00316-3.16 | 0.00316-3.16 | 0.00316-3.16 | 3.16-100 | 3.16-100 | 3.16-100 |
| **Slope** | 0.3054 | 0.6387 | 0.3046 | 0.6128 | 1.303 | 0.6083 |
| **0.95 CI** | 0.2594 to 0.3514 | 0.5837 to 0.6938 | 0.2684 to 0.3407 | 0.5313 to 0.6944 | 1.129 to 1.476 | 0.4941 to 0.7225 |
| **Dose-response** | linear | linear | linear | linear | linear | linear |
| **Number of doses** | 21 | 21 | 21 | 12 | 12 | 12 |
| **Number of subjects** | 21 | 21 | 21 | 21 | 21 | 21 |

## Figures

**Figure 1.3.1 Phase contrast image (10X) of Evaluation of cytotoxicity –**

**
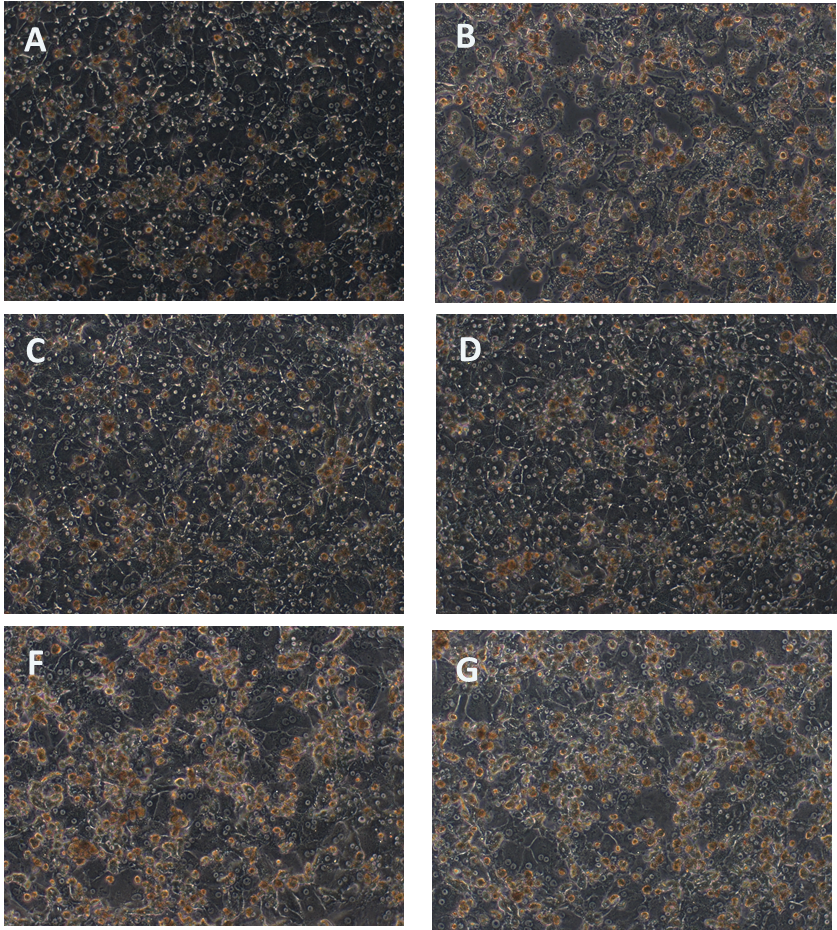
**

Morphological alterations indicative of cytotoxicity such as changes in cell shape, loss of cell‑cell contact, and accumulation of vacuoles, were monitored daily during treatment ([Guillouzo and Morel, 1997](#_ENREF_16)); ([Tyson and Green, 1987](#_ENREF_42)).. Hepatocyte Morphology following 72 hours of exposure to 0.1% DMSO (A), 50 µM Tamoxifen (positive control) (B), 100 µM CDCA (C), 100 µM OCA (D), 100 µM Tauro-OCA (E), and 100 µM Glyco-OCA (F). After 72 hours of CDCA or OCA exposure (C and D), no marked morphological changes were observed. Clear morphological changes were observed following exposure to 100 µM of tauro-OCA and glyco OCA (E and F).

**Figure 1.3.2. Dose linear response for mRNA expression of genes involved in bile acid synthesis and metabolism**


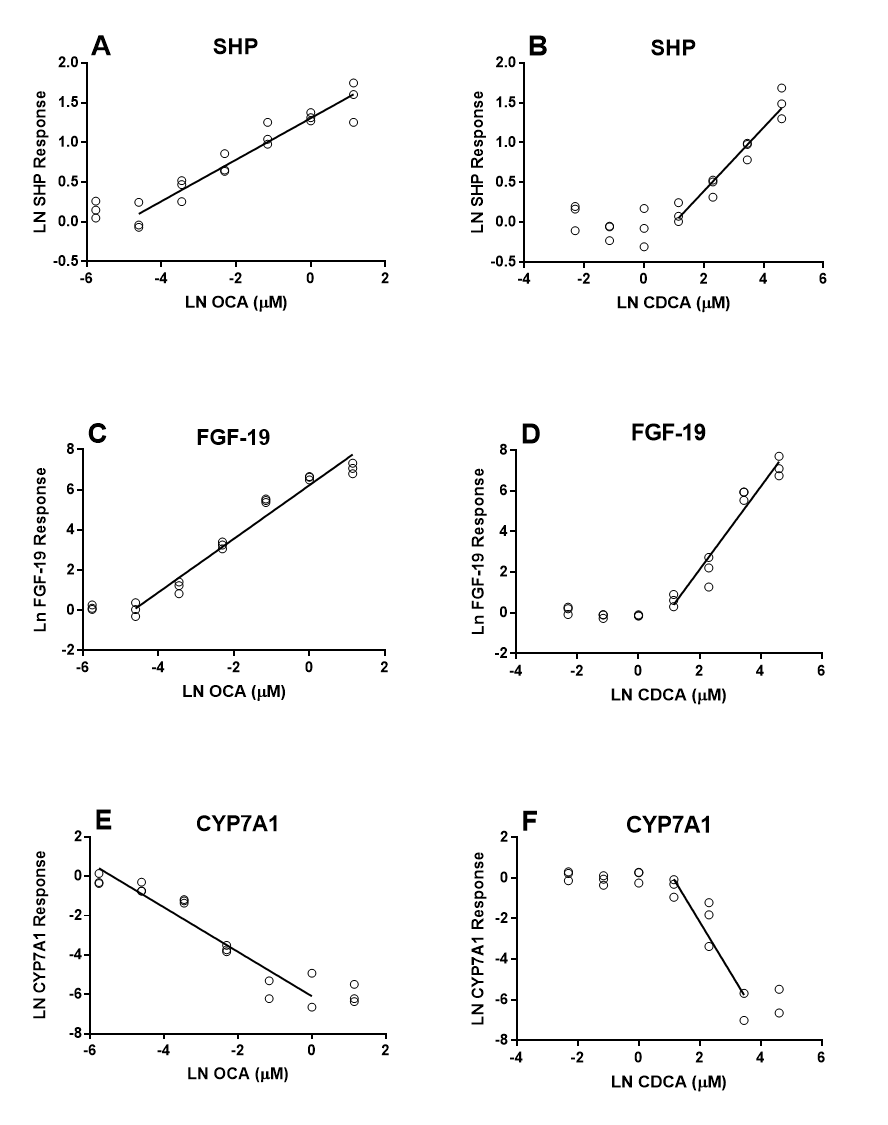


Data from in-text Figure 3 were used to construct dose-response profiles for SHP (A), FGF-19 (B), and CYP7A1 (C). Sandwich-cultured human hepatocytes from three donors were treated for 72 hours with CDCA (0.1, 0.316 1.0, 3.16, 10, 31.6, 100 µM) or OCA (0.00316, 0.01 0.0316, 0.1, 0.316, 1.0, 3.16 µM). Gene expression was determined using gene-specific TaqMan^®^ assays. PCR reactions were performed in triplicate wells for each donor and normalized to the vehicle control. The data represent individual dose responses from three donors. The solid line indicates the slope over the specified concentration range. Dose linear statistics are presented in Appendix Table 1.2.1

**Figure 1.3.3. Correlation response for mRNA expression of SHP versus CYP7A1 after exposure to OCA and CDCA**

Data from in-text Figure 3 were used to construct the correlation plots of SHP versus CYP7A1 after exposure to OCA (A) or CDCA (B). Sandwich-cultured human hepatocytes from three donors were treated for 72 hours with OCA (0.00316, 0.01 0.0316, 0.1, 0.316, 1.0, 3.16 µM) or CDCA (0.1, 0.316 1.0, 3.16, 10, 31.6, 100 µM). Gene expression was determined using gene-specific TaqMan^®^ assays. PCR reactions were performed in triplicate wells for each donor and normalized to the vehicle control. The data represent individual dose responses from three donors. Correlation statistics are presented in Appendix Table 1.2. 2.

**Figure 1.3.4. Measurement of mRNA expression of genes involved in bile acid synthesis and metabolism**


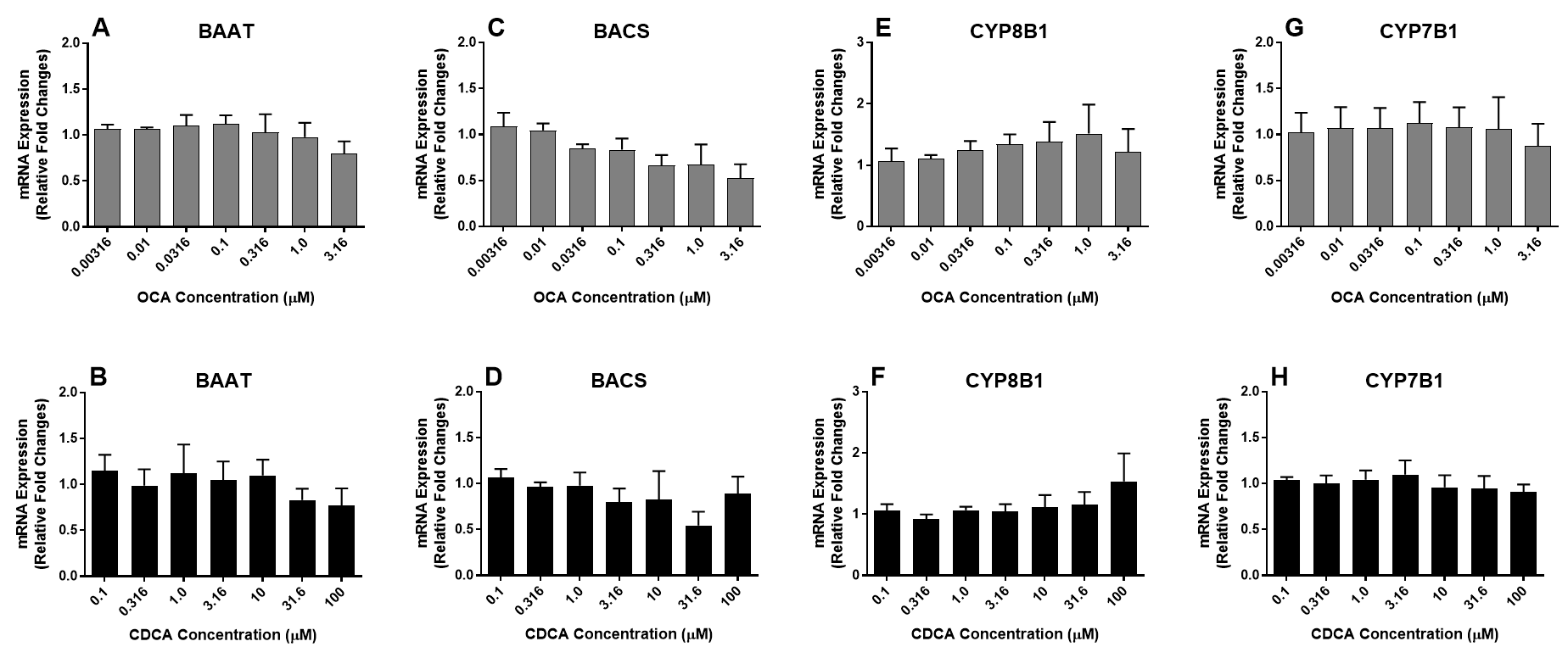


Sandwich-cultured human hepatocytes from three donors were treated for 72 hours with OCA panels A, C, E, and G at 0.00316, 0.01 0.0316, 0.1, 0.316, 1.0, 3.16 µM or CDCA panels B, D, F, and H at 0.1, 0.316 1.0, 3.16, 10, 31.6, 100 µM. BAAT (A, B), BACS (C, D), CYP8B1 (E, F), CYP7B1 (G, F), were evaluated following 72 hours of exposure to increasing concentrations of CDCA and OCA using gene-specific TaqMan^®^ assays. PCR reactions were performed in triplicate wells for each donor and normalized to the vehicle control. The data represent means ± SD from two donors for BAAT, BACS, CYP7B1, and from three donors for CYP8B1.

**Figure 1.3.5. Dose linear response of mRNA expression of bile acid transporters OST_α_, OST_β_, and BSEP** **in SCHH after treatment with OCA or CDCA**


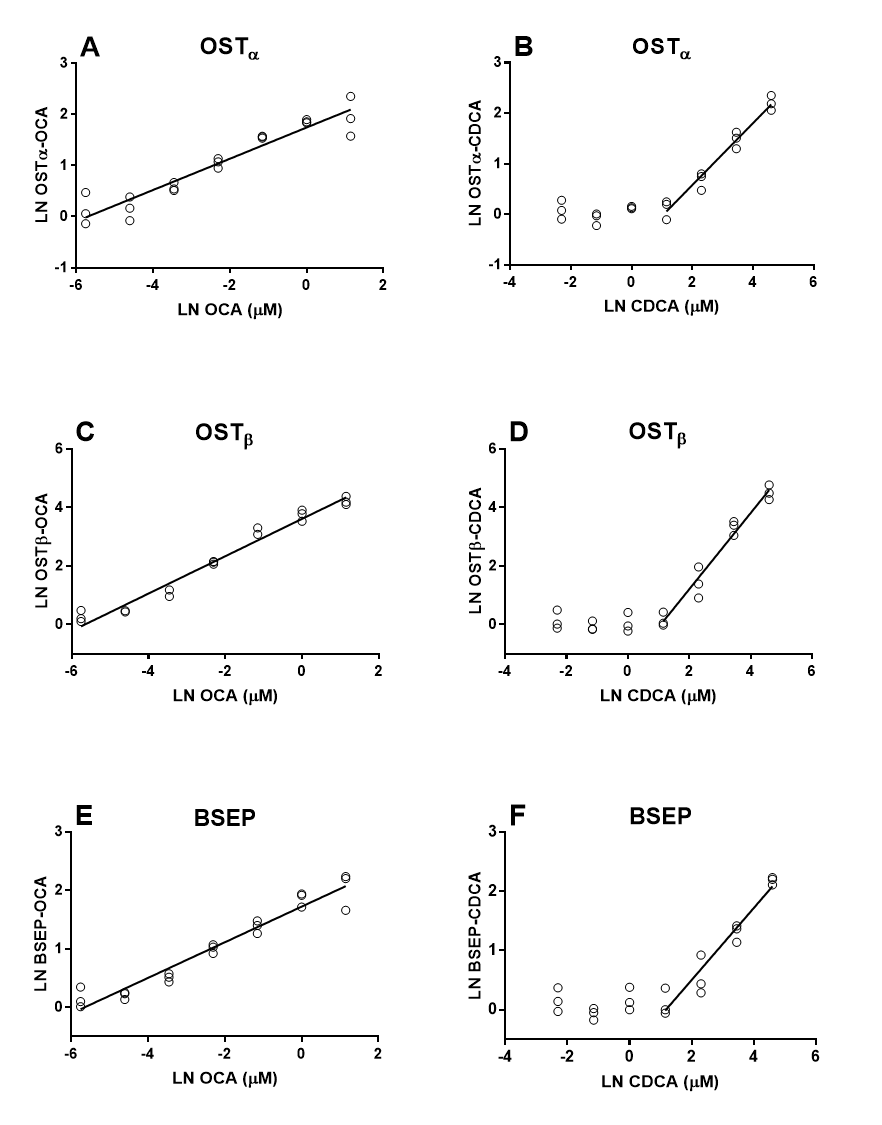


Data from in-text Figure 5 were used to construct dose-response profiles for OST_α_ (A and B), OST_β_ (C and D), and BSEP (E and F). Sandwich-cultured human hepatocytes from three donors were treated for 72 hours with OCA panels A, C, and E at 0.00316, 0.01 0.0316, 0.1, 0.316, 1.0, 3.16 µM or CDCA panels B, D, and F at 0.1, 0.316 1.0, 3.16, 10, 31.6, 100 µM. Gene expression was determined using gene-specific TaqMan^®^ assays. PCR reactions were performed in triplicate wells for each donor and normalized to the vehicle control. The data represent individual dose responses from three donors. The solid line indicates the slope over the specified concentration range. Dose linear statistics are presented in Appendix, Table 1.2.3.

**Figure 1.3.6. mRNA expression of bile acid transporters, NTCP, OATP1B1, OATP1B3, OATP2B1, P-gp, BCRP, MRP2, MRP3, and MRP4 in SCHH after treatment with OCA or CDCA**

**
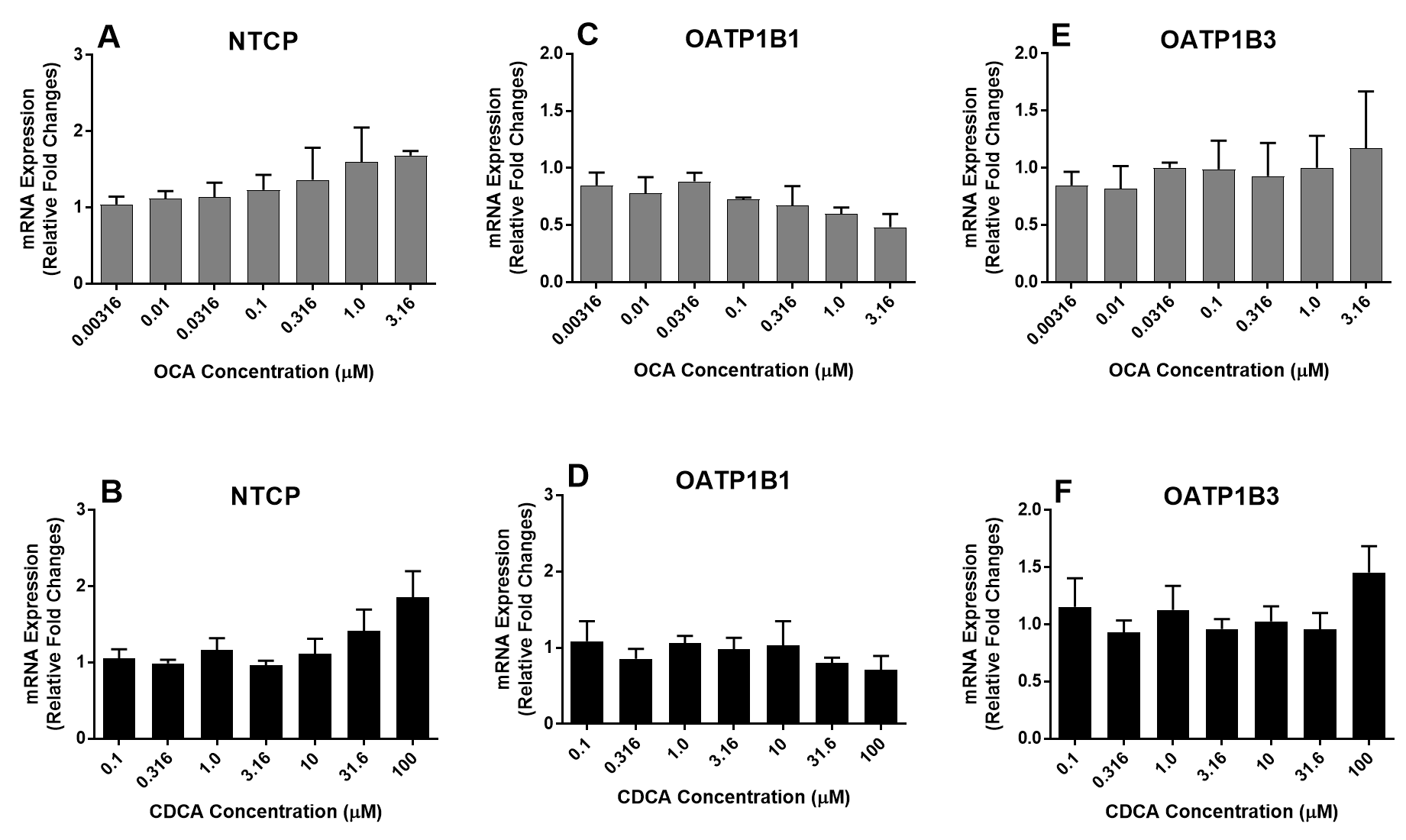
**

**
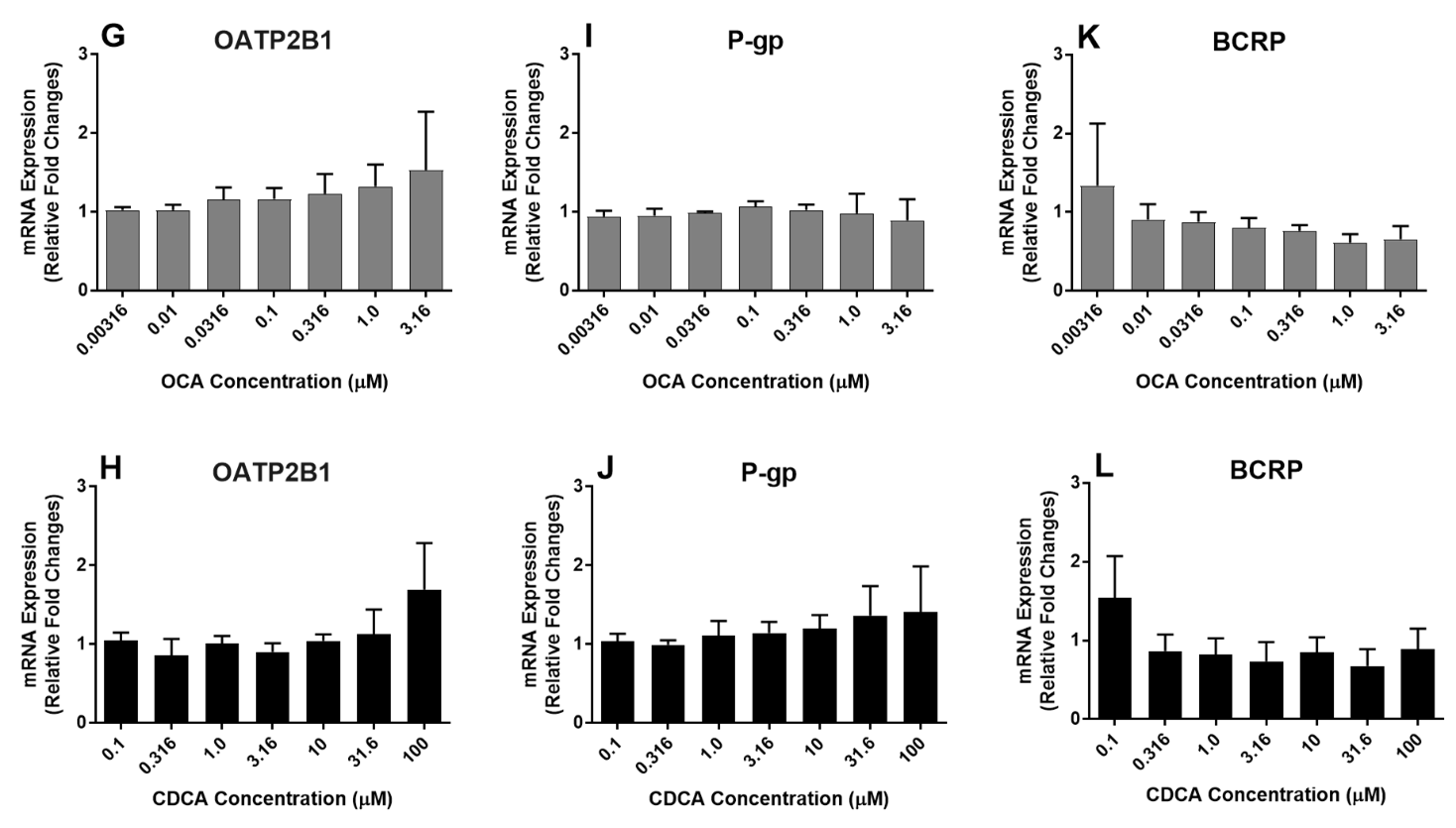
**

**
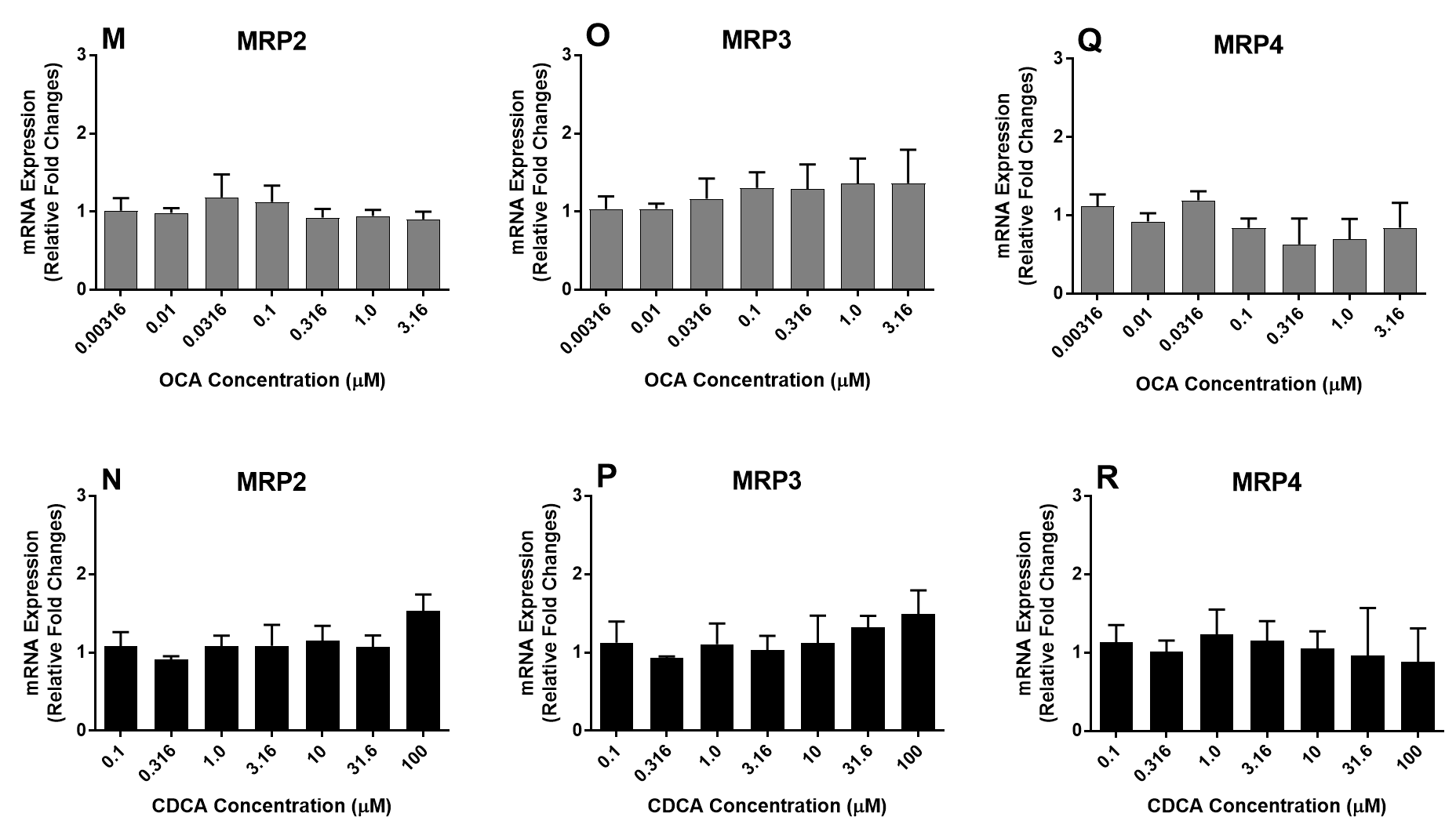
**

Sandwich-cultured human hepatocytes from three donors were treated for 72 hours with OCA panels A, C, E, G, I, K, M, O, and Q at 0.00316, 0.01 0.0316, 0.1, 0.316, 1.0, 3.16 µM or CDCA panels B, D, F, H, J, L, N, P, and R at 0.1, 0.316 1.0, 3.16, 10, 31.6, 100 µM. NTCP (A, B), OAT1B1 (C, D), OAT1B3 (E, F), OAT2B1 (G, H), P-gp (I, J), BCRP (K, L), MRP2 (M, N), MRP3 (O, P), and MRP4 (Q, R) were evaluated following 72 hours of exposure to increasing concentrations of CDCA and OCA using gene-specific TaqMan^®^ assays. PCR reactions were performed in triplicate wells for each donor and normalized to the vehicle control. The data represent means ± SD from two donors for BAAT, BACS, CYP7B1, and from three donors for CYP8B1.

**Figure 1.3.7: Gene expression comparison following treatments of OCA, glyco-OCA, tauro-OCA, and CDCA**

**
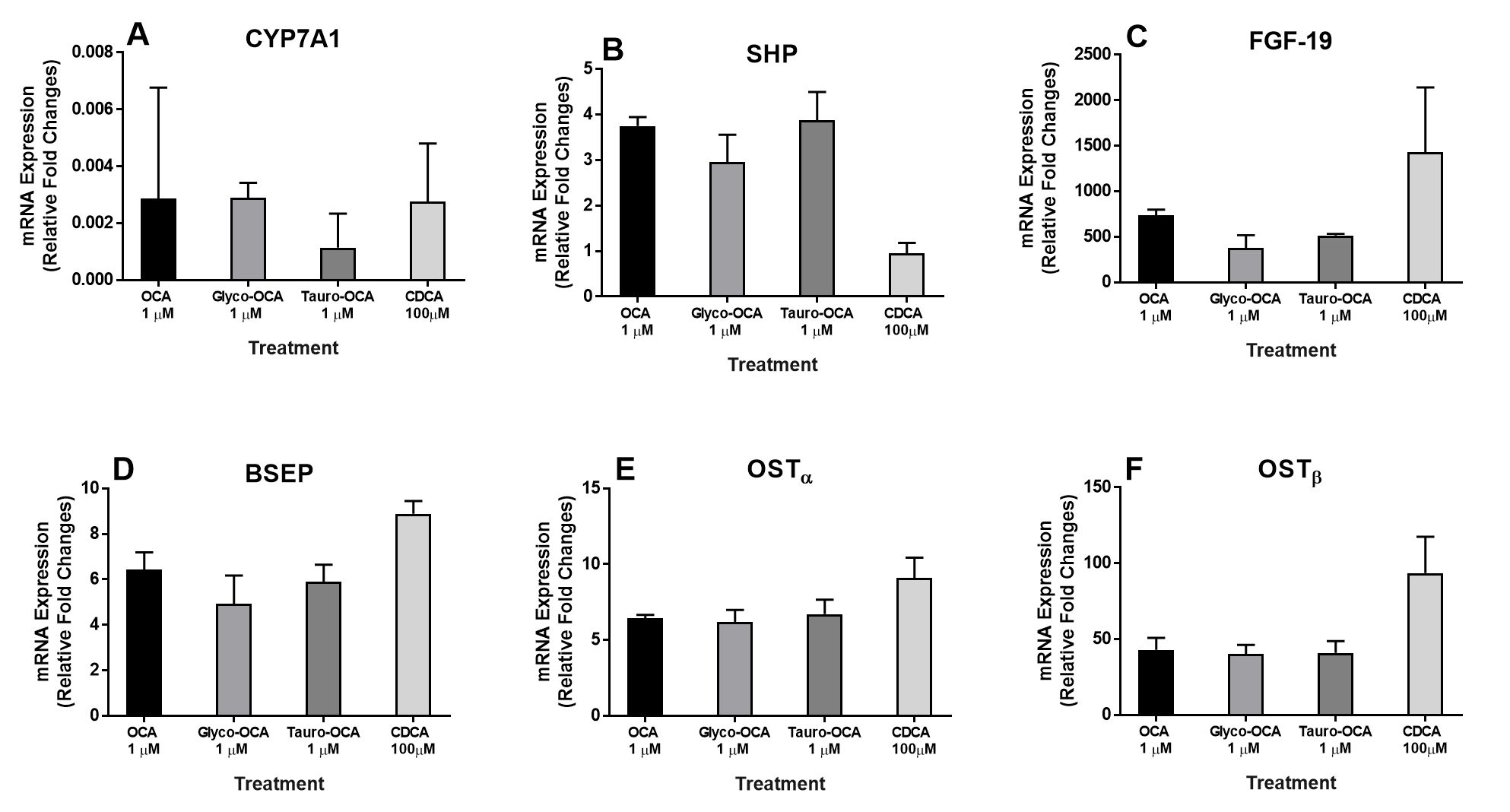
**

**
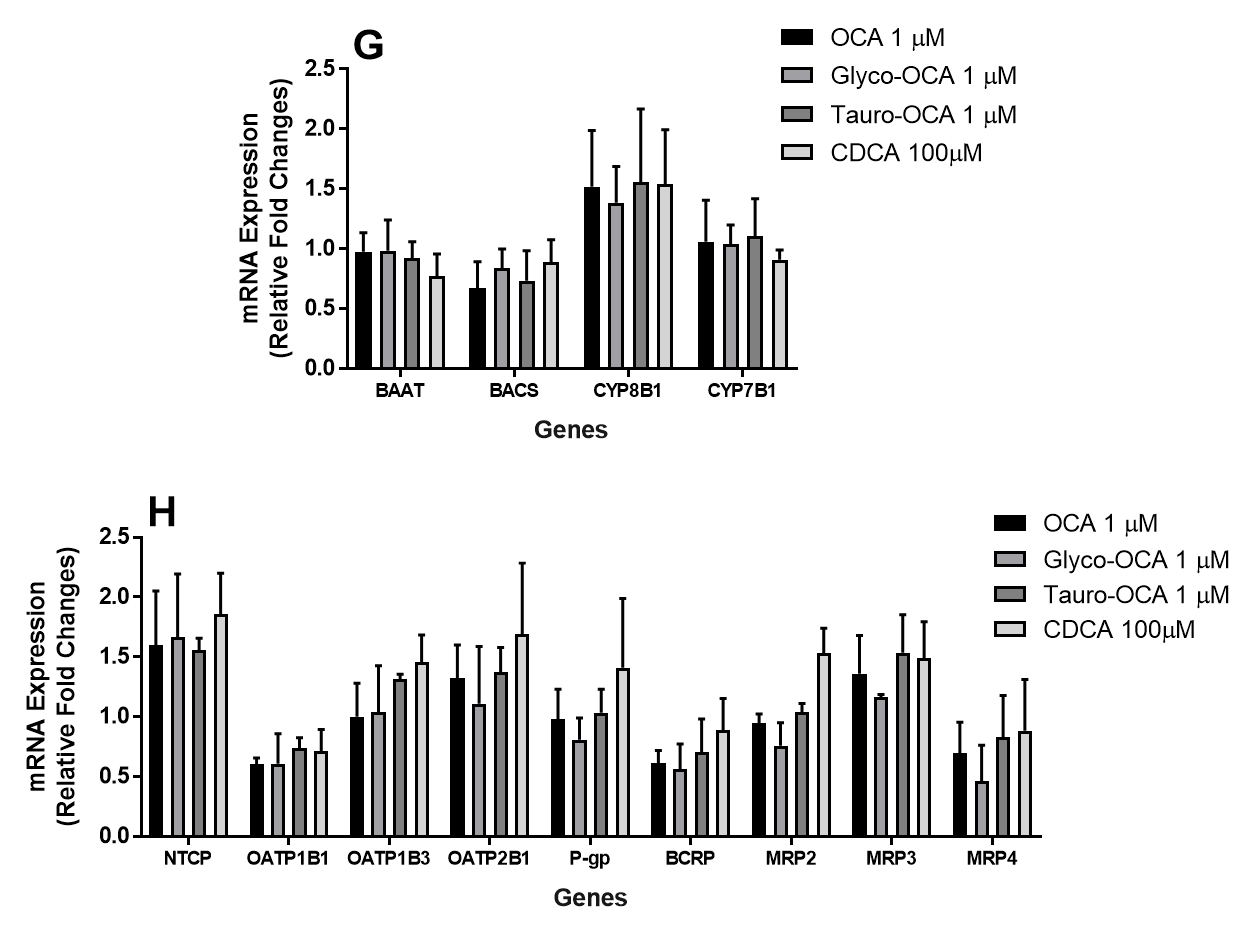
**

SCHH from three donors were treated for 72 hours with CDCA (0.1, 0.316 1.0, 3.16, 10, 31.6, 100 µM), OCA/glyco-OCA/ tauro-OCA (0.00316, 0.01 0.0316, 0.1, 0.316, 1.0, 3.16 µM). Genes: CYP7A1 (A), SHP (B), FGF-19 (C), BSEP (D), OSTα (E), OSTβ (F), BAAT, BACS, CYP8b1 and CYP7B1 (G) and NTCP, OATP1B1, OATP1B3, OATP2B1,P-gp, BCRP, MRP2, MRP3 and MRP4 (H) The dose-dependent effects of glyco-OCA and tauro-OCA on gene expression were very similar to OCA. To compare their effects, only data of 1 µM OCA/glyco-OCA/tauro-OCA and 100 µM CDCA are shown. The data represent means ± SD from three donors.
